# Feasibility and utility of active case finding of HIV-infected children and adolescents by provider-initiated testing and counselling: evidence from the Laquintinie hospital in Douala, Cameroon

**DOI:** 10.1186/s12887-018-1235-3

**Published:** 2018-08-03

**Authors:** Calixte Ida Penda, Carole Else Eboumbou Moukoko, Daniele Kedy Koum, Joseph Fokam, Cedric Anatole Zambo Meyong, Sandrine Talla, Paul Koki Ndombo

**Affiliations:** 1 Clinical sciences department, Faculty of Medicine and Pharmaceutical Sciences, University of Douala, PO Box 2071, Douala, Cameroon; 2 HIV Care and Treatment Centre, Laquintinie Hospital of Douala, Douala, Cameroon; 3 Virology Laboratory, Chantal Biya International Reference Centre for research on HIV/AIDS prevention and management, Yaoundé, Cameroon; 4 Faculty of Medicine and Biomedical Sciences, University of Yaoundé I, Yaoundé, Cameroon; 5 Technical office, Elizabeth Glaser Pediatric AIDS Foundation, LDH, Douala, Cameroon; 6 Mother-Child Centre, Chantal BIYA Foundation, Yaoundé, Cameroon

**Keywords:** HIV testing, Children, Adolescents, Entry points, MTCT, Cameroon

## Abstract

**Background:**

Universal HIV testing and treatment of infected children remain challenging in resource-limited settings (RLS), leading to undiagnosed children/adolescents and limited access to pediatric antiretroviral therapy (ART). Our objective was to evaluate the feasibility of active cases finding of HIV-infected children/adolescents by provider-initiated testing and counseling in a health facility.

**Methods:**

A cross-sectional prospective study was conducted from January through April 2016 at 6 entry-points (inpatient, outpatient, neonatology, immunization/family planning, tuberculosis, day-care units) at the Laquintinie Hospital of Douala (LHD), Cameroon. At each entry-point, following counseling with consenting parents, children/adolescents (0–19 years old) with unknown HIV status were tested using the Rapid Diagnostic Test (RDT) (Determine®) and confirmed with a second RDT (Oraquick®) according to national guidelines. For children less than 18 months, PCR was performed to confirm every positive RDT. Community health workers linked infected participants by accompanying them from the entry-point to the treatment centre for an immediate ART initiation following the « test and treat » strategy. Statistical analysis was performed, with *p* < 0.05 considered significant.

**Results:**

Out of 3439 children seen at entry-points, 2107 had an unknown HIV status (61.3%) and HIV testing acceptance rate was 99.9% (2104). Their mean age was 2.1 (Sd = 2.96) years, with a sex ratio boy/girl of 6/5. HIV prevalence was 2.1% (44), without a significant difference between boys and girls (*p* = 0.081). High rates of HIV-infection were found among siblings/descendants (22.2%), TB treatment unit attendees (11.4%) and hospitalized children/adolescents (5.6%); *p* < 0.001. Up to 95.4% (42/44) of those infected children/adolescents were initiated on ART. Overall, 487 (23.2%) deaths were registered (122 per month) and among them, 7 (15.9%) were HIV-positive; mainly due to tuberculosis and malnutrition.

**Conclusion:**

The consistent rate of unknown HIV status among children/adolescents attending health facilities, the high acceptability rates of HIV testing and linkage to ART, underscore the feasibility and utility of an active case finding model, using multiple entry-points at the health facility, in achieving the 90–90-90 targets for paediatric HIV/AIDS in RLS.

## Background

Paediatric HIV/AIDS remains a public health priority in children and adolescents worldwide, with 150,000 new infections occurring among children in 2015 [1], with over seventy-nine thousand (79,771) children and adolescents aged 0–19 years were living with HIV and almost half of them were 10 years and older during the same year [1, 2]. Most HIV-infected children are diagnosed late, at an advanced stage of disease progression [2, 3]. This programmatic challenge is of great concern because without antiretroviral therapy (ART), 53% of HIV-positive children die before their second birthday [4]. Thus, challenges in ensuring universal paediatric HIV testing and linkage to care are the driving force in reducing the gap between paediatric coverage and antiretroviral therapy (ART). Out the 1.8 million children living with HIV under the age of 15, only half are on ART worldwide, 20% of them in West and Central Africa in 2015 [5, 6]. Of note, at the time when paediatric ARVs were introduced in Cameroon in 2003, the number of HIV-infected children under 15 years was estimated at 50,334 and 50,284 for those in need of treatment. Though free access to ART (effective since 2007), coupled to progress in the WHO recommendations, has doubled the number of HIV-positive children accessing ART in Cameroon (3114 in 2007 to 6099 [11%] in 2014), the number children in need of ART (51,910 in 2014) remains very high, in the frame of a persistent paediatric HIV incidence nationwide (i.e. 4100 new infections reported in 2015) [7].

Several initiatives to scale up paediatric care have been implemented in recent years: i) the global Elimination Plan of MTCT in 2011 ii) the “Double Dividend” Initiative in 2013 through joint efforts of UNICEF, EGPAF and WHO with the dual goal of ending paediatric HIV epidemic and improving child survival in high HIV prevalence settings; and iii) “Accelerate Children’s HIV/AIDS Treatment Initiative” by PEPFAR and UNAIDS [8]. Cameroon has endorsed the UNAIDS strategic 90–90-90 targets: 90% of HIV-infected children and adolescents know their status, 90% of HIV-infected children who know their status are receiving ART and 90% of ART-experienced children have viral suppression [9]. Timely achievement of these targets requires implementing the “Test early, Test closer and Treat earlier” approach for every child and adolescent at all entry points of health facilities [8–10]. Successful implementation of this approach in linking to care warrants an assessment of HIV testing and access to ARVs for children/adolescents living with HIV and to improve quality of supply and demand for services. Our study objective was to ascertain the effectiveness of an active HIV case-finding model in HIV testing and linkages to care of children/adolescents at different entry points of a health facility in a RLS like Cameroon.

## Methods

### Study design

A cross-sectional and prospective study was conducted at the level of all 6 entry points of children and adolescents’ units of the Laquintinie hospital of Douala (LHD) in the Littoral region of Cameroon from January through April 2016. LHD has an Approved Treatment Centre (ATC) for HIV/AIDS day care where in 91% attendees are adults versus 9% children and adolescents. LHD is a centre of excellence for pediatric HIV care, with an active cohort of 452 children and adolescents that represents up to 32% HIV-infected children receiving ART in the Littoral region of Cameroon.

### Description of the study site

The HLD has 6 entry points of for pediatric care: (i) Pediatric inpatient unit that includes: Pediatric emergency, general pediatric hospitalizations, nutrition and sickle cell disease unit; (ii) Neonatology unit including premature babies and PMTCT services; (iii) Pediatric outpatient unit; iv) immunization and family planning unit; (v) Tuberculosis Screening and Management Unit (TB unit); and (vi) the day-care hospital/Approved treatment centre (ATC) for people living with HIV (PLWHIV): Adult unit and pediatric unit of care and treatment of PLWHIV. Regarding PMTCT, all of exposed HIV infant in our facility were managed in the PMTCT program. Nevirapine was administered for 6 weeks irrespective of the mode of feeding selected by the mother if she took ART (preferentially TDF + 3TC + EFV) for more than 1 month and for 12 weeks if the mother did not take ART or took them for less than one month.

### Sampling method

All children and adolescents aged 0–19 years, of unknown HIV status, or descendants of PLWHIV or siblings of HIV infected children, were consecutively by convenient sampling enrolled during the 4 months study period (January–April 2016). In the absence of real data on the prevalence of paediatric HIV infection for active HIV case finding in Cameroon, a minimum size for the study was calculated using the HIV prevalence of 15.4% based on a systematic review conducted on children and adolescents in sub-Saharan Africa [10], with z at 95% IC (i.e. z = 1.96) and an error rate of 0.05; to determine the minimum sample size using Cochran’s formula (z^2^*p*q/d^2^) [11], given a minimum of 201 children/adolescents for the study.

### Model of identification process and the patient flow

**Fig. 1:**
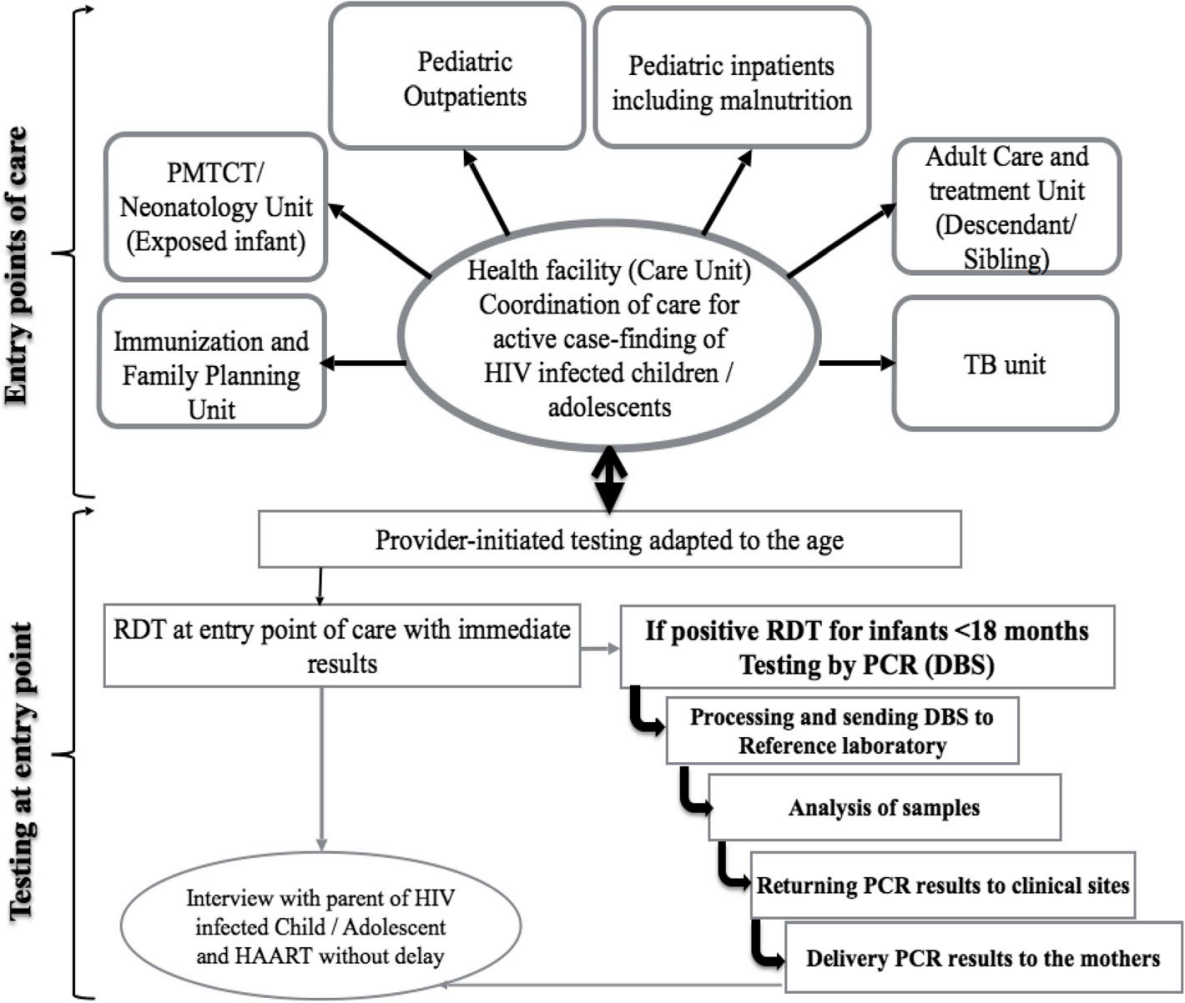
Identification model and screening of HIV for a child/adolescent at entry point of care in the central hospital level. DBS: dried blood spots; HAART: highly active antiretroviral therapy; HIV: human immunodeficiency virus; PCR: polymerase chain reaction; PMTCT: prevention of mother to child transmission of HIV; TB: tuberculosis; RDT: rapid diagnostic test

We developed a service model to actively look for cases of HIV-infected children and adolescents at all entry points at the LHD (Fig. 1). For every child and adolescent seeking care at any entry point of the LHD, we asked parents or guardians if the child’s HIV status was known and documented. If the answer was “no”, or “I don’t know”, information on the need for HIV testing was provided to parents by a trained community health worker (CHW) who provided pre-test counselling, written informed consent, onsite HIV testing and result delivery immediately after the post-test counselling. Additionally, all adult PLWHIV attending the ATC of the LHD were asked to take their descendant(s) aged 0–19 years whose HIV status was not known; for HIV-infected children attending the ATC, an active case finding of their siblings was also done. For descendants and siblings, study information was provided to parents during clinic attendance at the level of the waiting room, in order to enhance their motivation in bringing their family members for HIV testing. Then, a family tree of the descendants and siblings was developed from the index patient to determine the number of children and adolescents with unknown HIV status. The parent/guardian then decided on the location for HIV testing of the child or adolescent, who could be either at health facility or home-based. Additionally, HIV status of the mother was sought before carrying out the test of the neonate, infant and child.

The study pre-testing phase consisted of an administration of study tools (closed questions with single or multiple answers) to 20 parents/guardians over a period of one week in order to: (i) assess their understanding and acceptability and (ii) standardize and homogenize the data collection tools at the level of all entry points of the healthcare facility (Fig. 1).

### Procedure for HIV screening

**Fig. 2:**
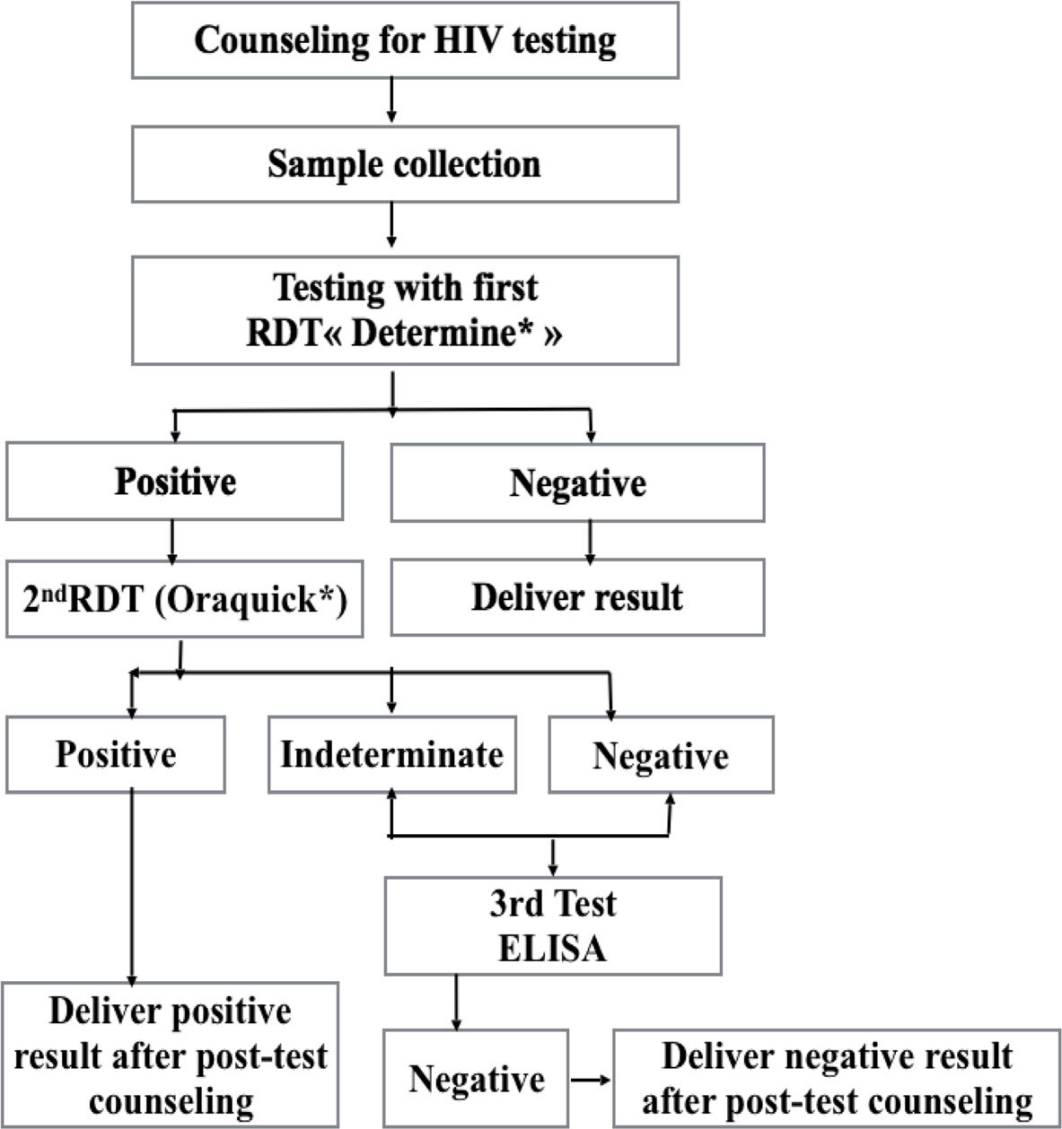
Screening Algorithm of HIV infection. ELISA: enzyme linked immunosorbent assay; HIV: human immunodeficiency virus; RDT: rapid diagnostic test

Based on a serial algorithm as per the national guidelines for HIV testing (Fig. 2), a rapid diagnostic test (RDT) was offered to consenting parents of participating children, with immediate result delivery, by task shifting from laboratory technicians to trained healthcare providers. Briefly, the first RDT (Determine™ HIV-1/2) was performed using capillary blood from the child/adolescent as per the manufacturer’s instructions, with a sensitivity of 100% (98.5–100) and a specificity of 95.8% (93.3–98.4) for HIV-1/2 evaluated locally [12]. After 15 min, the result was provided and post-test counselling done accordingly. In case of a non-reactive HIV result, the child/adolescent has declared HIV-negative (i.e. free of HIV-infection); in case of a reactive HIV result, a second more specific RDT (Oraquick®) was performed as per the manufacturer’s instructions, with a sensitivity of 96.7% (94.4–98.9) and a specificity of 100% (98.5–100) to confirm HIV infection, as per local assessment [12]. In case of discordant results between the two RDTs, an ELISA test (ELISA” Genscreen™ ULTRA HIV Ag - Ab) was performed as tier breaker following the manufacturer’s instructions (i.e to confirm or exclude HIV-infection). Post-test counselling was provided prior to result delivery. In case of a reactive HIV result after RDT in an infant < 18 months, a blood sample was collected from a prick on the heel, toe or finger, directly into a filter paper (Whatman n° 903) for a confirmation of the result by polymerase chain reaction (PCR). Of note, the HIV status of the mothers was sought before carrying out the test of the neonate, infant or child. For HIV-vertically exposed infants of less than 18 months, the serological test reflects the exposure to HIV through their mothers, which requires testing by PCR to either confirm or infirm HIV-infection. For any HIV-positive child/adolescent, CHWs accompanied the concerned from the entry point to the ATC for an immediate initiation on ART according to the strategy of «test and treat». The parents /legal guardians were provided with therapeutic education by a psychosocial agent. Children tested HIV-positive at inpatient services were also initiated on ART and monitored throughout their hospitalization (Fig. 2).

### Statistical analyses

Categorical variables were expressed as frequency, while the quantitative variables were presented as means ± Standard deviations (SD) or with 95% interval confidence (IC 95%) if normally distributed. To compare proportions, we used Fisher exact test. Quantitative values were compared using the U-test of Wilcoxon test. Only variables with a *p*-value ≤0.2 in the univariate model were considered for analysis in a multivariate logistic regression model. All statistical analyses were performed using the Stata (version 11SE) and R (version 3.1.1 software). *P*-value < 0.05 was considered statistically significant.

## Results

### Basic characteristics and acceptability of HIV testing among study participants

**Fig. 3:**
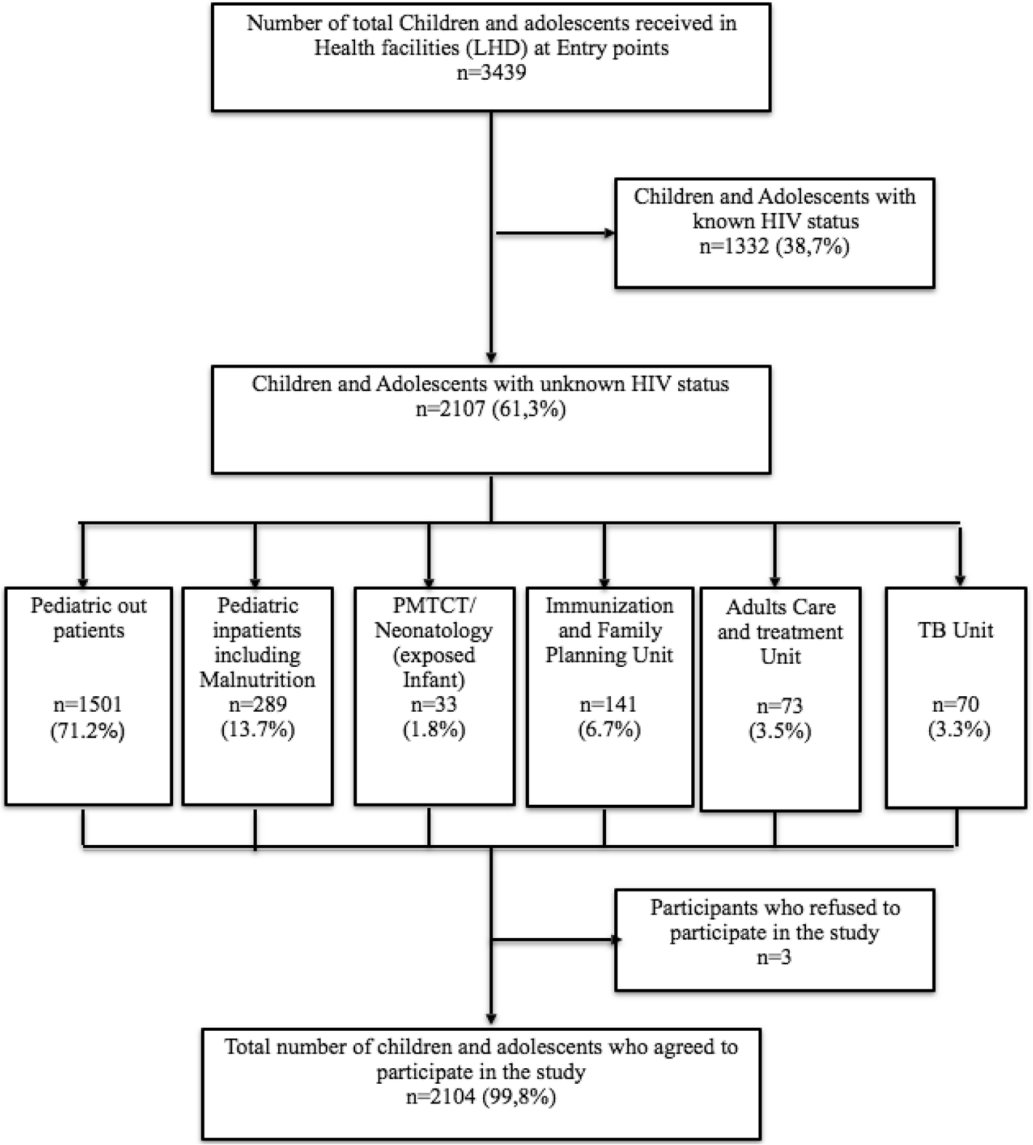
Flow diagram of child/adolescent enrolled in the Study. HLD: Laquintinie hospital of Douala; HIV: human immunodeficiency virus; PMTCT: prevention of mother to child transmission of HIV; TB: tuberculosis

Overall, 3439 interviews were conducted to parents/legal guardians of children/adolescents attending the LHD, and up to 2107 children/adolescents were reported to have an unknown HIV status, indicating a rate of 61.3% unknown HIV infection in this paediatric population (Fig. 3).

Three parental refusals of consent were recorded, among which one each from the in-patient unit, outpatient unit and family tree model, giving 99.9% (2104/2107) acceptability rate for enrolment and HIV testing in the entire study population (Fig. 3).

Among 2104 children and adolescents enrolled, the majority came from outpatient unit (71.29%), followed by hospitalized patients (13.69%), as shown in Table 1. Immunization and family planning unit contributed 6.69% of children and adolescents. A comparison of acceptability based on the six entry-points showed no significant difference in performance (*p* = 0.090).

**Table 1:** Basic characteristics of the study population by sex and age range

Variables	Girls	Boys	Total	*P*
Number of participants enrolled: n (%)	953 (45.3)	1.151 (54.7)	2.104 (100.0)	
Entry point of care				
Pediatric Inpatients Unit^*^	116 (12.2)	172 (14.9)	288	1
Pediatric out patient Unit	679 (71.2)	821 (71.3)	1.500	0.121
TB Unit	37 (3.9)	33 (2.8)	70	0.056
Vaccination and Family Planning Unit	71 (7.4)	70 (6.1)	141	0.048
PMTCT/Neonatology Unit	21 (2.2)	12 (1.0)	33	0.010
Adult care and treatment Unit (Descendant/Sibling)	29 (3.0)	43 (3.7)	72	0.898
Mean Age (Sd), years	2.06 (2.95)	2.13 (2.96)	2.10 (2.96)	0.329
[0–3]^a^	756 (79.3)	875 (76.0)	1.633 (77.5)	1
[3–6]	95 (10.0)	154 (13.4)	249 (11.8)	0.015
[6–9]	49 (5.1)	67 (5.8)	116 (6.5)	0.391
[9–11]	22 (2.3)	18 (1.6)	40 (1.9)	0.354
[11–15]	25 (2.6)	31 (2.7)	56 (2.7)	0.801
[15–19]	6 (0.6)	6 (0.5)	12 (0.6)	0.801

Data are number and/or proportion (%), unless otherwise indicated; *PMTCT* prevention of mother to child transmission of HIV, *SD* standard deviation, *TB* tuberculosis; ^a^: Reference

Overall, among 2104 children and adolescents included boys accounted for 54.71% and the boy/girl sex ratio was 6:5 (1151/953) and the mean age of 2.10 (Sd = 2.96) years. The age class 3–6 years of boys was significantly higher compared to that of girls (15.0% vs. 11.2% for girl, *p* = 0.015) (Table 1). No difference was observed between the number of girl and boys patients according to entry point of care. However, the mean age was significantly higher among children enrolled from the family tree model (5.47 +/-Sd = 4,33) and from the tuberculosis (TB) unit (4.81 + / Sd = 4,50 years), as compared to that of children/adolescents enrolled from in-patient services, (*p* = 0.0001).

### HIV prevalence and linkage to ART care according to entry points

Out of 2104 children and adolescents tested for HIV, 44 were diagnosed as HIV-positive, giving an overall prevalence of 2.1% (Table 2). According to entry points, the highest rates of HIV-infection were reported among siblings/descendants, TB unit attendees and hospitalized patients, respectively with 22.2, 11.4 and 5.6%, with statistically significant differences among participants from siblings/descendants, outpatient unit, immunization and family planning (*p* ≤ 0.001). Of note, none (0%) of the 141 infants enrolled from the immunization unit was positive, and only 2 (0.1%) of the 1500 children enrolled from outpatient unit were HIV-positive.

**Table 2:** Distribution of population according to HIV status at different entry points of care

Entry point of care	HIV status, n (%)			
	HIV Negative 2060 (97.9)	HIV Positive 44 (2.09)	Total 2104	*P*
Pediatric inpatients Unit^a^	272 (94.4)	16 (5.56)	288	1
Out Pediatric patient Unit	1498 (99.9)	2 (0.1)	1500	< 0.0001
TB Unit	62 (88.6)	8 (11.4)	70	0.078
Immunization and Family Planning Unit	141 (100.0)	0	141	0.004
PMTCT/Neonatology unit	31 (93.9)	2 (6.1)	33	0.905
Adult care and treatment Unit (Descendant/Sibling)	56 (77.6)	16 (22.2)	72	< 0.0001

Data are number and/or proportion (%), unless otherwise indicated; ^a^: Reference; *TB* Tuberculosis, *PMTCT* Prevention of mother to child transmission of HIV

Among the 44 HIV positive children and adolescents, 42 (95.5%) were successfully accompanied by CHWs to the ATC and were all enrolled into care according to the national guidelines for pediatric management of HIV/AIDS. Thus, this gives a very high linkage to care among HIV-positive children/adolescents, with only 4.5% refusal. Prior to ART initiation, two died during hospitalization for malnutrition and TB. Among the 40 HIV-infected children/adolescents who effectively initiated on ART, 5 died subsequently, giving an overall mortality rate of 15.9% (7/44) of HIV positive children/adolescents enrolled in the study (Table 3). In contrast, within the population of HIV-negative children, up to 23.2% (480) mortality rate was reported mainly due to life threatening paediatric emergencies (Table 3). This gives an overall mortality rate of 23.2% (487), resulting to 122 deaths per month.

**Table 3:** Mortality in the population of HIV-infected and uninfected children /adolescents

Survival status	Patients						
	HIV Infected		HIV Uninfected		Total		
	n	%	n	%	n	%	*P*
Died	7	15.9	480	23.2	487	23.2	1
Alive	37	84.1	1580	76.8	1617	76.8	0.85

*HIV* human immunodeficiency virus, *n* number; %: proportion

## Discussions

In order to contribute to the global efforts for ending AIDS, we designed and implemented a strategy for universal HIV testing and enrolment to care of all infected children/adolescents in RLS. Our model of multiple entry points to healthcare was highly accepted by parents/legal guardians (> 99%), similar to findings from Uganda (92.8%) and Kenya (82.5%) [11, 13, 14]. These indicate a high success rate of such approach in RLS, which contributes in achieving the 90% HIV diagnosis among children/adolescents with unknown status [9]. In this frame, Provider Initiated Testing and Counselling (PITC) for the children are feasible.

Our model of active linkage to care, using CHWs for liaison persons, showed an excellent enrolment into care of HIV-positive children/adolescents (> 95%). Thus, the current model, if well implemented, would contribute in achieving 90% of ART coverage in HIV-infected children/adolescents [9].

Routinely, HIV testing is offered to suspect children or those under the PMTCT program, both accounting for only 10% of children attending consultation and those admitted to the hospital. Of note, the prevalence was low in PMTCT/Neonatology due to maternal exposure to ART, except for children of HIV-infected mother who missed the PMTCT program and were discovered at delivery or postnatal unit (Neonatology). Moreover, task shifting of HIV testing to non-health professionals (CHW), under guardian/parental counselling, significantly reduces waiting time and increases access/acceptability to testing [15].

The mean age of our study population was 2.1 years, higher than those in Bamenda-Cameroon (1.3 year) and in Zambia (1 year) [16, 17]. Nonetheless, these observations altogether indicate late HIV diagnosis in RLS, with high mortality among infected children if untreated [15]. In this model, there is need for ensuring earlier diagnosis and treatment in order to limit HIV-associated mortality [18, 19]. This is crucial for siblings/descendants with unknown HIV status, who often have very late diagnosis (5.47 years in our finding). As previously reported, using family tree as a key to identify unreported children living with HIV and increase paediatric ART would be relevant [10, 15, 20, 21].

HIV prevalence from our study was lower (~ 2%) than those from Malawi and Uganda but higher than that of Kenya [13, 15, 16, 19]; disparities being mainly attributed to varying epidemics in these different geographical settings. HIV prevalence varies by entry point, with a high burden at the TB Unit (11.4%), similar to findings from Ethiopia (14.5%) [22]. TB unit should be considered as a secondary point to catch-up missing cases of paediatric HIV for linkage to care in RLS [22, 23], thus closing the gap in paediatric ART coverage (~ 40 increased fold in Uganda) [24].

The rate of HIV-associated mortality (15.9%) was similar to those in West and Central Africa (16%) in 2014 [25]. However, the high mortality (23.2%) among HIV-negative children was due to late hospital attendance with life threatening emergencies in the frame of malnutrition, TB and encephalopathy. Of note, as a referral centre, the LHD as a referral centre receives cases with clinical complications from primary healthcare facilities and with higher risk of mortality, thereby justifying the surprisingly high mortality rates among HIV-negative children.

A major strength of our findings is the high sensitivity of Determine (100%) used as first RDT and the high specificity of Oraquick (100%) used as second RDT, as reported by Njouom et al. in Cameroon [12], indicating accuracy in identifying the real serological status. However, studies on costing of the current model, that integrates community-based HIV-testing, linkage to care and viral load coverage in pediatric populations, would provide further evidences for policy-making toward ending paediatric AIDS in RLS [1, 9].

## Conclusion

A model of HIV testing of children/adolescents at multiple entry points and active linkage to care is feasible and efficient in achieving universal paediatric ART coverage in African RLS. With emphasis on family tree, TB, and/or hospitalised children/adolescents, this model would greatly contribute in achieving the current global targets for paediatric HIV in Cameroon and in other RLS.

## Abbreviations

AIDS: Acquired immunodeficiency syndrome
ART: Antiretroviral therapy
ATC: Approved Treatment Centre
CHW: Community health workers
EGPAF: Elizabeth Glaser Pediatric AIDS Foundation
HIV: Human immunodeficiency Virus
LHD: Laquitinie Hospital of Douala
PCR: Polymerase Chain Reaction
PEPFAR: US President Emergency Fund for AIDS Relief
PLWHIV: People Living with HIV
PMTCT: Prevention of Mother-to-Child Transmission
RDT: Rapid Diagnostic Test
RLS: Resource-Limited Settings
TB: Tuberculosis
UNAIDS: Joint United Nations Programme on HIV/AIDS
UNICEF: United Nations Children’s Fund
WHO: World health organization
